# Expression Profiling and Molecular Modeling Analysis of *Cyp51C* 14α-Demethylase Associated with Azole Resistance in Clinical *Aspergillus flavus* Isolates

**DOI:** 10.3390/jof12070466

**Published:** 2026-06-25

**Authors:** Ines Hadrich, Nahed Khemakhem, Houaida Trabelsi, Hayet Sellami, Moez Elloumi, Fattouma Makni, Ali Ayadi, Sourour Neji

**Affiliations:** 1 Fungi and Parasitic Molecular Biology Laboratory, School of Medicine, University of Sfax, Sfax 3029, Tunisia; nahedkhemakhem@hotmail.com (N.K.); thouaida@yahoo.fr (H.T.); hayetsellami1@gmail.com (H.S.); famakni@gmail.com (F.M.); ali.ayadi@rns.tn (A.A.);; 2 Laboratory of Parasitology—Mycology, UH Habib Bourguiba, Sfax 3029, Tunisia; moez.elloumi@rns.tn

**Keywords:** antifungal, *A. flavus*, PCR-sequencing, Cyp51, RT-qPCR

## Abstract

Invasive infections caused by *Aspergillus flavus* are more common in tropical and subtropical countries. The emergence of azole resistance in *A. flavus* complicates the management of aspergillosis, as azoles are the first-line and empirical therapy. The aim of this study was to investigate the molecular mechanisms underlying azole resistance in *A. flavus*, focusing on the *cyp51C* gene. We screened 34 molecularly confirmed *A. flavus* isolates obtained from patients with invasive aspergillosis for *cyp51C* gene expression by real-time RT-qPCR and for mutations by PCR sequencing. Molecular modeling and docking studies were performed using SWISS-MODEL, SwissDock, and I-TASSER software. Susceptibility testing revealed that 14.71% and 8.82% of isolates were resistant to itraconazole and posaconazole, respectively, with 5.88% exhibiting cross-resistance. The mRNA expression of *cyp51C* was upregulated (>2.5-fold) in five of the six resistant strains (83.33%). Hyperexpression of *cyp51C* was significantly more frequent among resistant isolates than among susceptible isolates (Fisher’s exact test, *p* = 0.014). Sequencing identified ten point mutations, including six synonymous and four non-synonymous substitutions. The non-synonymous mutations M54T and S240A were detected in the protein sequences of both resistant and susceptible isolates. Notably, D254N and I285V were observed exclusively in resistant isolates and in susceptible isolates with itraconazole MICs near the epidemiological threshold. Homology modeling and 3D structure prediction of the mutated Cyp51C protein demonstrated interactions with itraconazole, posaconazole, and voriconazole. Importantly, I-TASSER analysis indicated that the I285V substitution is located near the itraconazole binding site. Simultaneous overexpression of the *cyp51A*, *cyp51B* and *cyp51C* genes was observed in 33.33% of resistant isolates. These findings suggest that multiple target genes and mechanisms may act concurrently to confer azole resistance in *A. flavus*. Overall, this study supports the hypothesis that azole resistance in *A. flavus* is multifactorial and highlights the potential value of combining mutation analysis, gene expression profiling, and structural modeling for improved molecular surveillance and antifungal resistance monitoring.

## 1. Introduction

Aspergillosis is a major cause of morbidity and mortality in immunocompromised patients, primarily due to delayed immune recovery or difficulties in early diagnosis [1]. The type and severity of disease are influenced by both the physiological state of the patient and the infecting *Aspergillus* species. Azole antifungals remain the mainstay for prophylaxis, treatment of acute infections, and long-term management of chronic and invasive aspergillosis [2]. However, the widespread clinical use of azoles has led to the emergence of azole-resistant aspergillosis, including multiple-triazole resistant strains, which are associated with poor prognosis and high mortality [3,4,5]. The increasing emergence of azole-resistant isolates represents a major clinical challenge, emphasizing the need to better understand the molecular mechanisms underlying antifungal resistance in clinically relevant *Aspergillus* species.

Azoles, including itraconazole, posaconazole, and voriconazole, act by inhibiting ergosterol biosynthesis through 14α-demethylases (Cyp51 proteins), which are encoded by *cyp51* genes. Unlike other *Aspergillus* species, which encode two Cyp51 proteins (Cyp51A and Cyp51B), *A. flavus* harbors three Cyp51 proteins: Cyp51A, Cyp51B and Cyp51C. This unique genetic configuration suggests a more complex regulatory network controlling azole susceptibility in *A. flavus* [4,6]. In addition, azole resistance is increasingly recognized as a multifactorial phenomenon involving not only alterations in *cyp51* genes but also the coordinated regulation of ergosterol biosynthesis pathways, stress-response mechanisms, and other cellular adaptive processes.

Although the molecular mechanisms underlying azole resistance in *A. flavus* are not fully understood, multiple genes and mechanisms are increasingly recognized to act simultaneously in conferring resistance [7,8]. Several studies have highlighted the contribution of *Cyp51C* to resistance. For instance, substitutions S196F and N423D were reported to significantly affect the structural conformation and drug binding of *Cyp51C* [7], and the T788G missense mutation in the *cyp51C* gene was associated with voriconazole resistance in *A. flavus* [4]. Furthermore, point mutations in *cyp51A* or *cyp51C* have been linked to the increased expression of these genes relative to susceptible strains [8]. Nevertheless, the precise contribution of cyp51C alterations to azole resistance remains insufficiently characterized, particularly in comparison with the extensively studied *cyp51A*-mediated resistance mechanisms.

Based on these observations, the present study aimed to elucidate the mechanisms of azole resistance in *A. flavus* isolates by investigating, first, mutations and expression of the *cyp51C* gene, and second, the interactions and binding of azole antifungals to the active site of the Cyp51 enzyme. By integrating gene expression analysis, mutation profiling, and structural modeling, this study also seeks to contribute to the identification of molecular markers that are potentially useful for resistance surveillance and improved antifungal management of aspergillosis.

## 2. Material and Methods

### 2.1. Patients and Isolates

The population of 34 molecularly confirmed *A. flavus* sensu stricto clinical isolates included in the present study was collected from 14 patients hospitalized in the Haematology Unit, Hedi-Chaker Hospital, Sfax, Tunisia. Weekly samples (sputum and nasal swabs) were obtained from immunocompromised patients with invasive aspergillosis. Eighteen isolates were cultured from sputum, 10 from nasal swabs, 5 from bronchoalveolar lavage, and 1 from a pulmonary biopsy. All *A. flavus* strains were cultured on Sabouraud dextrose agar (SDA; AES). Identification of the isolates was based on the morphological characteristics and DNA sequencing of the rRNA gene’s internal transcribed spacer (ITS) regions [9]. Antifungal susceptibility was determined using the ETEST method after 48 h of incubation, as previously described [10]. Epidemiological cutoff values (ECVs) were defined as 0.25 µg/mL for posaconazole (POS) and 1 µg/mL for itraconazole (IT) and voriconazole (VOR) has been described previously by five laboratories, as determined by the CLSI M38-A2 microdilution method at 48 h [11].

### 2.2. Mechanisms of Azole Resistance

DNA and RNA were extracted using the QIAamp DNA Mini Kit (QIAGEN, Hilden, Germany) and the RNeasy Mini Kit (Qiagen^®^, Hilden, Germany), respectively, following the manufacturer’s protocols. Reverse transcription (RT) was carried out using the PrimeScript™ RT Reagent Kit (Perfect Real Time) from TaKaRa (Shiga, Japan). Specific primers and probes for gene expression analysis (Table 1) and PCR sequencing (Table 2) were designed using the OligoArchitect online primer design tool (Sigma-Aldrich, St. Louis, MO, USA).

#### 2.2.1. Quantitative Real-Time PCR (qPCR)

The expression level and copy number of the cyp51C gene were determined by quantitative real-time PCR (qPCR), with normalization against the housekeeping gene Actin (ATC).

Each reaction consisted of 10 μL of TaqMan Universal PCR Master Mix, 1 μL of template (DNA or cDNA), 20 pmol of primers, and 7 pmol of hydrolysis probe. All assays were performed in triplicate using the StepOne™ Real-Time PCR system (Applied Biosystems, Knutsford, UK). Relative quantification (RQ) values were calculated using StepOne™ software version 2.1 (Applied Biosystems, UK) according to the following formula:

RQ = 2 − [Cq Target − Cq Reference] Tested − [Cq Target − Cq Reference] Control

A 2.5-fold variation was considered indicative of gene overexpression or increased gene copy number [12,13]. All qPCR reactions were performed in triplicate.

#### 2.2.2. Sequencing of PCR Products

The *A. flavus cyp51C* gene (NCBI accession no. XM_002383890.1) was amplified as three overlapping fragments (*cyp51CP1*, *cyp51CP2* and *cyp51CP3*). PCR reactions were carried out in a thermocycler (Eppendorf, Hamburg, Germany) in a final volume containing 10 μL of 5× reaction buffer, 25 mM MgCl_2_, 0.2 mM of each dNTP (dATP, dCTP, dGTP, and dTTP; Promega, Hampshire, UK), 20 pmol of each primer, 2.5 U of GoTaq^®^ DNA polymerase (Promega, UK), and 400 ng of genomic DNA.

Amplicons were purified using the Wizard^®^ PCR Purification Kit (Promega, UK) and sequenced with the BigDye^®^ Terminator Cycle Sequencing Kit v3.1 (Applied Biosystems, UK). Sequence analyses were performed by comparison with reference wild-type *A. flavus cyp51C* sequences using the NCBI BLASTN tool (https://blast.ncbi.nlm.nih.gov/Blast.cgi) (accessed on 18 May 2026) and ClustalW 2.1. (https://www.genome.jp/tools-bin/clustalw, accessed on 18 May 2026).

### 2.3. Molecular Modeling

The DNA sequences of *cyp51C* genes for 34 strains of *A. flavus* (6 resistant (R) and 28 sensitive (S) to azoles) were submitted to NCBI GenBank database with the access number presented in Table 3. Then, they were translated into the equivalent amino acid sequences by using the Geniegen2 software (https://www.pedagogie.ac-nice.fr/svt/productions/geniegen2/, accessed on 18 May 2026). The BLAST server (NCBI, Bethesda, MD, USA) was used for Protein Data Bank (PDB) similarity searches to find homologous sequences (models) that matched known experimental 3D structures [14]. To align the protein sequences, the Clustal Omega program server (EMBL-EBI, Hinxton, UK) was used https://www.ebi.ac.uk/jdispatcher/msa/clustalo (accessed on 18 May 2026) and the automated protein structure homology modeling server SWISS-MODEL (SIB, Lausanne, Switzerland) generated a homology model for each target protein https://swissmodel.expasy.org/ (accessed on 18 May 2026) [15].

The crystal structures of sterol 14-alpha demethylase (Cyp51B) from a pathogenic filamentous fungus *A. fumigatus* in complex with different triazole deposited in the PDB under accession number 4UYL, 5FRB and 6CR2 were used as the template.

Molecular docking studies were performed using the open-access docking server (PatchDock) (https://www.cs.tau.ac.il/~ppdock/PatchDock/) (accessed on 18 May 2026) [16]. PatchDock identifies potential molecular interactions based on geometric shape complementarity between two molecules, including proteins, DNA, peptides, and small ligands. The generated docking solutions were ranked according to shape complementarity criteria and subsequently submitted for refinement and rescoring using the FireDock web server (https://bioinfo3d.cs.tau.ac.il/FireDock/) (accessed on 18 May 2026). The receptor–ligand complexes generated by PatchDock were further refined using FireDock. The refined complexes were ranked according to their binding energies, and 3D structural visualizations were generated to facilitate the analysis and comparison of molecular interactions [17].

To further investigate the interaction between the mutated Cyp51C protein and the antifungal agents evaluated in this study, three-dimensional protein modeling was performed based on crystal structure prediction. Then, we used the I-Tasser software (https://zhanggroup.org/I-TASSER/) (accessed on 18 May 2026). Indeed, this server is a powerful tool allowing for the fast, accurate and reliable prediction of the three-dimensional structure of our protein, which is modified without using homology modeling. This structure, in the form of a PDB file, will be used as an input file for the molecular docking software.

The I-TASSER server output for each given sequence includes up to five full models, the estimated confidence score, TM score and RMSD, as well as the deviation type of estimates. During our study, we also used this software to search and predict the binding sites of different ligands on our target protein.

Molecular docking is an empirical method that allowed us to predict the interaction between our Cyp51C protein and the three azoles used during our study. For this, we used the SwissDock software (https://www.swissdock.ch/) (accessed on 18 May 2026). Visualization and analysis of the docking poses were carried out using UCSFChimera molecular viewer (University of California, San Francisco, CA, USA).

From the 3D Cyp51C protein structural model predicted by I-Tasser, the COFACTOR program (https://zhanggroup.org/COFACTOR/) (accessed on 18 May 2026) chained the request through the BioLiP protein function database (https://zhanggroup.org/BioLiP/) (accessed on 18 May 2026) through correspondence between local and global structures in order to identify the binding sites of the ligands of our Cyp51C protein. The predicted binding pockets were subsequently used for molecular docking analyses. At the end of the docking procedure, a Protein Data Bank (PDB) file containing the receptor–ligand complex structure was generated for further visualization and interaction analysis.

### 2.4. Statistical Analyses

Statistical analyses were performed using Fisher’s exact test to evaluate associations between *cyp51C* hyperexpression and azole resistance phenotypes due to the limited sample size and low expected frequencies. A *p*-value < 0.05 was considered statistically significant.

Relative expression levels between resistant and susceptible isolates were compared using the non-parametric Mann–Whitney U test.

## 3. Results

### 3.1. Mechanisms of Azole Resistance

#### 3.1.1. Levels of *Cyp51C* Expression by *A. flavus* Isolates

The expression level of the *cyp51C* gene was evaluated relative to the housekeeping gene ACT1, used as a stable endogenous control, and to the highly susceptible reference strain *A. flavus* TN-855 (minimum inhibitory concentration (MIC) IT: 0.032 μg/mL; MIC VOR: 0.025 μg/mL; MIC POS: 0.012 μg/mL; GenBank accession number JX852588).

Total RNA from 34 clinical *A. flavus* isolates (28 susceptible strains, 3 ITC-resistant strains, 1 POS-resistant strain, and 2 strains exhibiting cross-resistance to IT and POS) was extracted and reverse-transcribed into cDNA. Quantitative real-time PCR (qPCR) was then performed to determine both the relative expression level and the copy number of the *cyp51C* gene in each isolate.

**Figure 1:**
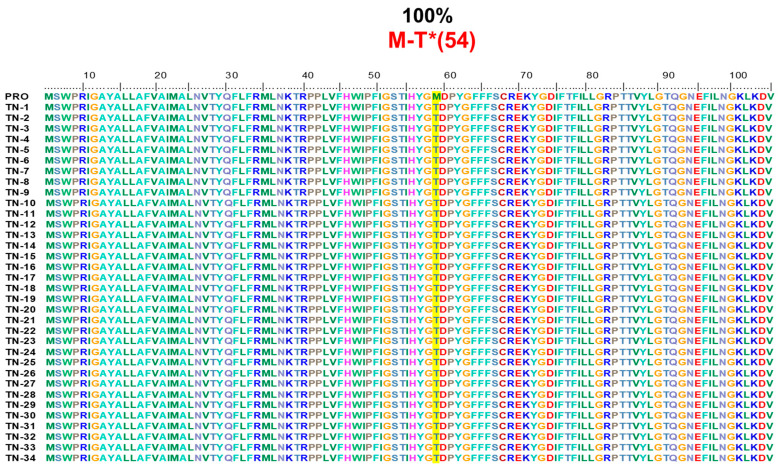

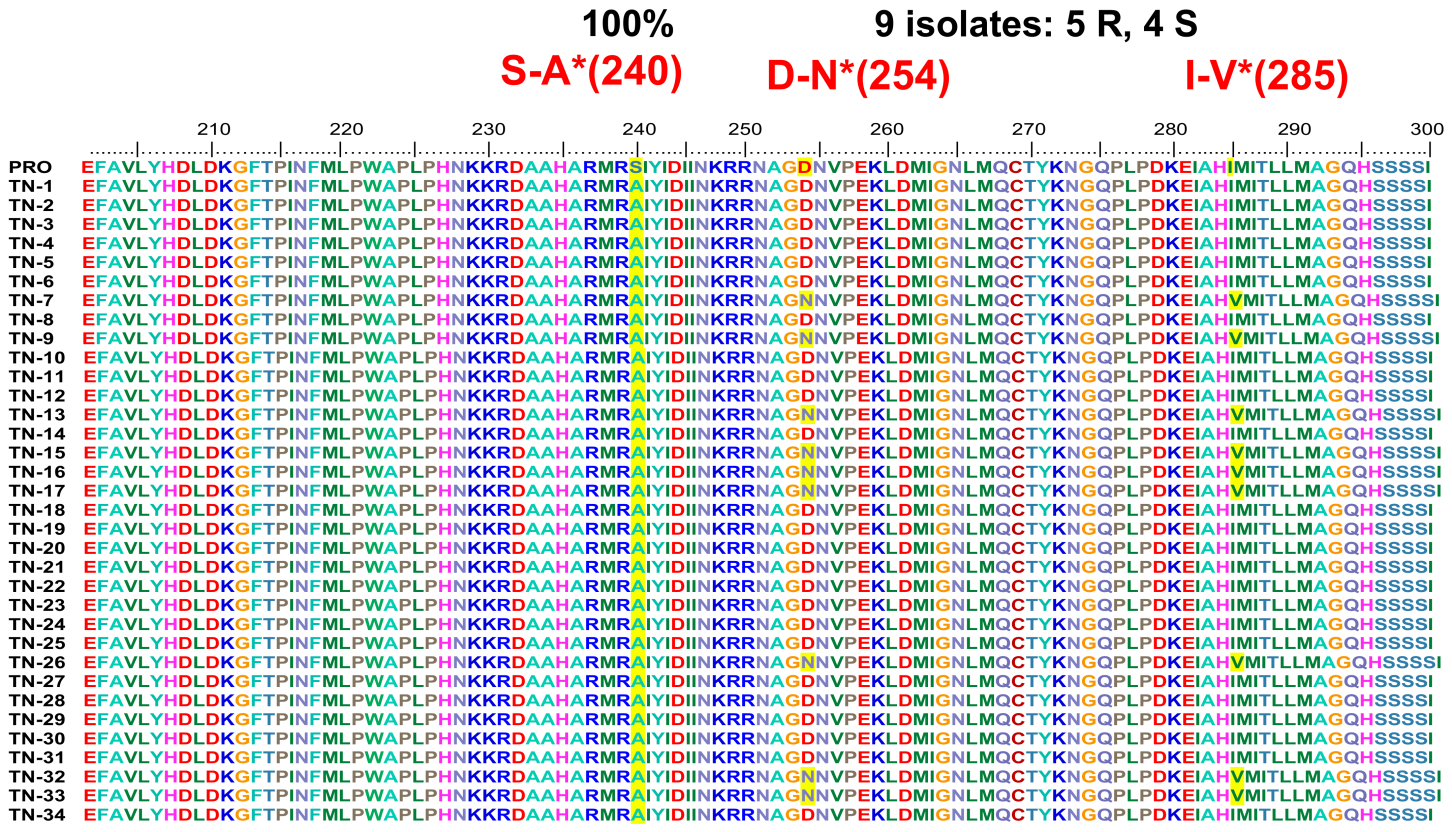
Alignment of Cyp51C protein sequences from *A. flavus* was compared with wild-type *A. flavus* strain (Genbank ID: XP_002383931.1). Colored residues represent detected amino acid substitutions. R indicates resistant isolates and S indicates susceptible isolates according to antifungal susceptibility testing. All isolates carried the M54T and S240A substitutions. In addition, nine isolates (five resistant and four susceptible isolates) harbored the D254N and I285V substitutions. * Punctual mutation.

Gene expression analysis revealed hyperexpression exceeding a 2.5-fold threshold relative to ACT1 and the reference strain TN-855. Overall, hyperexpression of *cyp51C* was detected in 12 of the 34 clinical isolates (35.29%), including both susceptible and resistant strains. Expression levels ranged from 0.027- to 2.54-fold across all isolates (Figure 1, Table 2).

Among susceptible isolates, seven strains exhibited hyperexpression, with comparable expression levels observed in TN-4, TN-10, and TN-12. A marked increase in *cyp51C* expression was observed in strain TN-20 and in strain TN-26, the latter presenting an IT MIC of 0.75 μg/mL.

Among resistant isolates, five of six strains (83.33%) showed *cyp51C* overproduction. The highest expression level (5.474; corresponding to a 2.18-fold increase) was detected in the IT/POS cross-resistant strain TN-33, followed by the IT-resistant strain TN-15 (2.14-fold). Hyperexpression of *cyp51C* was significantly more frequent among resistant isolates (5/6; 83.33%) than among susceptible isolates (7/28; 25%) (Fisher’s exact test, *p* = 0.014).

An increased copy number of the *cyp51C* gene was observed in 5 of 34 isolates (14.7%). Only one IT-resistant isolate (TN-16) exhibited a significant increase in gene copy number (3.952). Multiple copies of *cyp51C* were also detected in four susceptible isolates (11.76%).

Overall, hyperexpression of *cyp51C* was observed in most resistant isolates, except for strain TN-7, which did not exhibit significant overexpression.

Based on the study conducted by Ghorbel et al. on the same *A. flavus* population investigating *cyp51A* and *cyp51B* overexpression, four distinct expression profiles involving the three *cyp51* genes encoding enzymes of the ergosterol biosynthesis pathway were identified among resistant isolates [18].

The IT/POS cross-resistant strain TN-31 exhibited higher expression levels of *cyp51A* (5.19-fold) and *cyp51B* (4.52-fold) compared with *cyp51C* (1.02-fold). The IT/POS-resistant strain TN-33 showed hyperregulation of *cyp51A* (6.75-fold) and *cyp51C* (2.18-fold) without *cyp51B* overexpression, suggesting a possible contribution of these two genes to azole resistance.

In strain TN-32 (POS-resistant), *cyp51B* and *cyp51C* expression levels were comparable (3.51- and 3.649-fold, respectively), whereas a pronounced increase in *cyp51A* expression (7.49-fold) was detected. Resistant isolates exhibited significantly higher median *cyp51C* expression levels compared with susceptible isolates (*p* < 0.05).

Strains TN-16 and TN-15, both showing reduced IT susceptibility, exhibited isolated *cyp51C* hyperproduction (1.97- and 2.14-fold, respectively), suggesting that resistance in these isolates may be associated with *cyp51C* expression independently of *cyp51A* and *cyp51B*.

Interestingly, the IT-resistant strain TN-7 did not display upregulation of any of the three cyp51 genes, indicating that alternative resistance mechanisms may be involved.

Simultaneous overexpression of *cyp51A*, *cyp51B* and *cyp51C* was observed in 2 resistant isolates (33.33%), whereas no susceptible isolate exhibited concurrent hyperexpression of the three genes.

#### 3.1.2. Detection of Point Mutations in the *cyp51C* Gene

Multiple sequence alignment of the obtained *cyp51C* sequences with the reference *A. flavus cyp51C* sequence (NCBI accession number XM_002383890.1) revealed six synonymous (C-G*(174), G-A*(757), G-A*(781), T-C*(946), T-A*(964), C-T*(1228)) and four non-synonymous point mutations (T-C* (161), T-G*(788), G-A*(830), A-G*(923)) (Table 3). The synonymous point mutations identified in this study appear to be novel, whereas the four non-synonymous mutations have been previously described.

The four non-synonymous mutations resulted in the following amino acid substitutions: M54T, S240A, D254N, and I285V (Figure 1).

Substitutions M54T and S240A were detected in all 34 isolates regardless of their susceptibility profiles and were therefore considered neutral polymorphisms without apparent phenotypic impact.

In contrast, substitutions D254N and I285V were identified in IT-resistant isolates (TN-7, TN-15, TN-16; MIC > 1 μg/mL), in the IT/POS cross-resistant strain TN-33, in the POS-resistant strain TN-32, and in several susceptible isolates (TN-17, TN-13, TN-26, TN-9) presenting IT MIC values (0.75 μg/mL) close to the epidemiological cutoff according to the CLSI M38-A2 reference method.

### 3.2. Molecular Modeling

**Figure 2:**
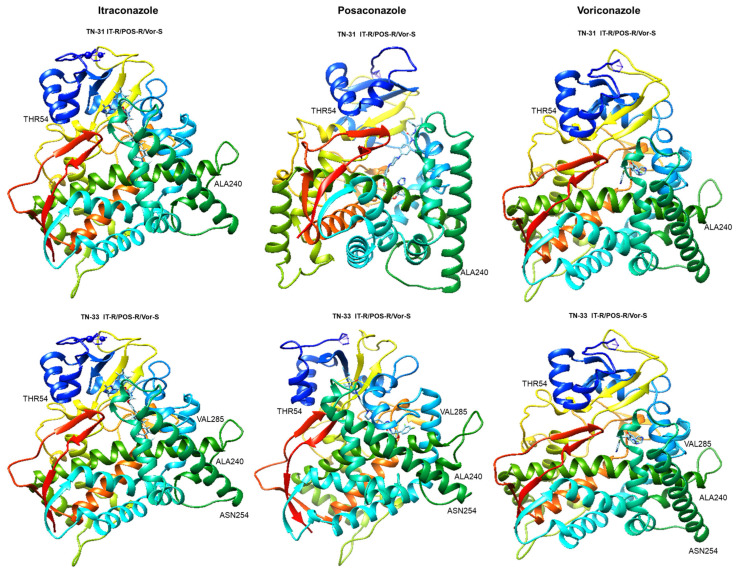
Overall view of the 3D model of the *A. flavus* Cyp51C protein in complex with three antifungals (IT, POS and VOR). S—susceptible; R—resistant. The positions of the amino acid substitutions are indicated. Colors were automatically assigned by UCSF Chimera to facilitate visualization of the protein secondary structure and do not represent any specific physicochemical property.

Structural modeling and docking analysis performed using UCSF Chimera indicated that the non-synonymous substitutions M54T, S240A, D254N, and I285V did not alter the overall structural integrity of the Cyp51C protein. Furthermore, all three azole antifungals (IT, VOR, and POS) were able to interact with the mutated enzyme structure (Figure 2).

**Figure 3:**
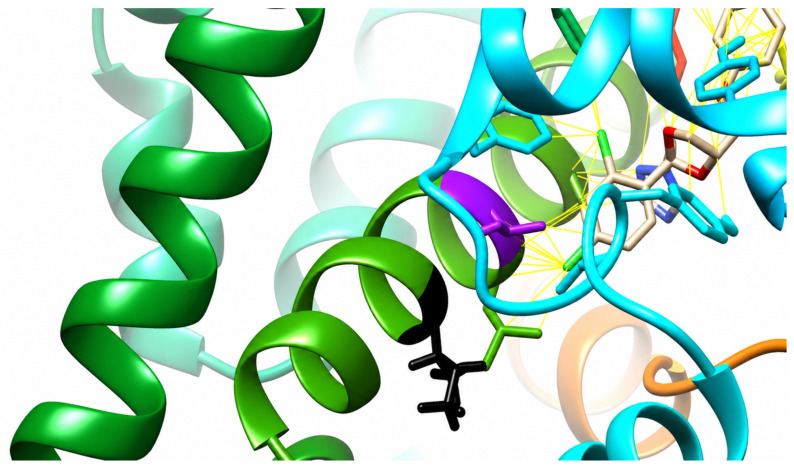
Itraconazole binding site in our Cyp51C protein, created using the I-Tasser program. The protein structure is shown as a ribbon representation. Itraconazole is shown as black sticks, amino acid residues involved in binding are represented as colored sticks, and yellow lines indicate predicted intermolecular interactions.

Ligand-binding site prediction using the COFACTOR program (https://zhanggroup.org/COFACTOR/, accessed on 18 May 2026), based on the I-TASSER structural model, revealed that residue I285 is located in close proximity to the azole-binding site (IT and POS), positioned two amino acids away from threonine 288, which participates in ligand interaction (Figure 3, Table 4).

The substitution of isoleucine by valine at position 285 (I285V) may therefore subtly modify the local active-site environment, potentially reducing ligand affinity and antifungal efficacy. This structural observation may explain the presence of this substitution in resistant isolates as well as in susceptible isolates exhibiting IT MIC values (0.75 μg/mL) near the epidemiological cutoff.

Accordingly, the resistance observed in strain TN-7 may be partially associated with the I285V substitution in the *cyp51C* gene.

The resistant phenotype may involve the combined contributions of *cyp51* paralog overexpression and structural alterations.

## 4. Discussion

*A. flavus*, the second leading cause of IA, is widely distributed in the environment, including the soil, water, and air [19,20,21]. Recent epidemiological studies indicate that *A. flavus* represents a major etiological agent of IA in several geographic regions, particularly in arid and subtropical areas, where its prevalence may equal or even exceed that of *A. fumigatus* in specific endemic regions [22,23]. Individuals with severely impaired immune systems are prone to IA after inhaling *A. flavus* spores. The increasing clinical use of azole antifungals to treat *A. flavus* infections raises concerns regarding the emergence and selection of azole-resistant strains. Recent surveillance data have reported an increasing proportion of *A. flavus* isolates exhibiting elevated minimum inhibitory concentrations (MICs) to triazoles, emphasizing the growing clinical relevance of resistance in this species [24,25,26].

In order to study azole drug resistance in *Aspergillus* species, the three genes encoding 14-α sterol demethylase enzymes (*cyp51A*, *cyp51B* and *cyp51C* genes) were considered [18]. Our investigation was focused on the third paralog, *cyp51C*, as a key difference between *A. flavus* and other *Aspergillus* species [4].

The analysis of *cyp51C* expression in the 34 isolates demonstrated that overexpression was detected in the majority of resistant strains, although not universally present, highlighting heterogeneity in resistance mechanisms. Importantly, *cyp51C* hyperexpression was significantly more frequent among resistant isolates than among susceptible isolates (Fisher’s exact test, *p* = 0.014), supporting a statistical association between *cyp51C* overexpression and reduced azole susceptibility. However, our previous results reported differences in the expression levels of *cyp51A* and *cyp51B* in the same population [18]. In the present study, we showed the simultaneous overexpression of the three genes *cyp51A* and *cyp51B* and *cyp51C* in two (33.33%) of the resistant isolates. No sensitive strain showed hyperexpression of three genes at the same time. These findings support the hypothesis that azole resistance in *A. flavus* may result from cumulative effects involving multiple *cyp51* paralogs rather than a single dominant resistance mechanism [27,28]. Importantly, the absence of uniform overexpression among resistant isolates further supports the multifactorial nature of resistance in this species.

Studies on the resistance mechanisms have shown that amino acid residue substitution derived from mutations in the azole target enzyme gene *cyp51A*, overexpression of this gene and drug efflux genes, and upregulation of homeostatic stress response pathways contribute to azole resistance in *A. fumigatus* [3,29,30]. In contrast, resistance mechanisms in *A. flavus* appear more heterogeneous, with increasing evidence supporting a modulatory role of *cyp51C* sequence polymorphisms and expression variability [6,25].

In order to study azole drug resistance in *A. flavus*, the three genes encoding 14-α sterol demethylase enzymes (*cyp51A*, *cyp51B* and *cyp51C* genes) were sequenced in all *A. flavus* strains included in this study and their deduced amino acid sequences were compared. We used the sequence of the *A. flavus* type strain (NRRL3357) as the reference sequence. The use of NRRL3357 as a reference strain is supported by its complete genome annotation and its widespread use in molecular and resistance studies [31].

The *cyp51C* gene exhibited polymorphism, with several synonymous and non-synonymous point mutations detected among both susceptible and non-susceptible isolates. The presence of these substitutions across different susceptibility profiles suggests that most represent naturally occurring polymorphisms rather than direct determinants of resistance. Non-synonymous mutations found in this study were previously described, such as M54T, D254N, I285V and S240A [6], while other substitutions are novel. However, the presence of M54T and S240A in both susceptible and non-susceptible strains indicates they are neutral polymorphisms likely associated with geographical or population-level variation, rather than direct antifungal selection pressure [27]. Notably, the S240A substitution, previously linked to voriconazole resistance through site-directed mutagenesis [4], was ubiquitous in our collection, aligning with recent evidence that it is a naturally occurring polymorphism without phenotypic consequence [32].

Lui et al. demonstrated by gene mutagenesis of the cyp51C gene in *A. flavus* NRRL3357 that the non-synonymous point mutation T788G, associated with amino acid substitution at serine 240 (S240), contributed to resistance to VOR [4]. However, subsequent studies demonstrated that this mutation is also present in susceptible isolates, indicating that on its own it is not sufficient to confer azole resistance [6,25].

The amino acid substitutions M54T and S240A were detected in all 34 isolates regardless of their susceptibility profiles and were therefore considered neutral. Such polymorphisms likely represent lineage-associated or naturally occurring population variations rather than resistance-associated mutations [26].

Structural modeling using I-TASSER showed that isoleucine 285 is located close to the ligand-binding site, near threonine 288, which is involved in azole interaction. Consequently, the I285V substitution may induce subtle conformational changes affecting the local drug-binding environment. However, docking analyses did not demonstrate major alterations in global protein structure or complete disruption of ligand interaction, suggesting that any potential effect is likely moderate and indirect. These in silico predictions should be interpreted with caution in the absence of functional validation.

In addition, Paul et al. reported that the Y319H mutation, although far from the iron-porphyrin complex, can act indirectly on drug binding [6]. This supports the concept that remote substitutions in *cyp51C* may exert long-range structural effects. Accordingly, the I285V substitution may contribute to altered susceptibility through subtle structural modulation rather than acting as a primary resistance determinant.

Interestingly, strain TN-7 exhibited resistance without detectable overexpression of *cyp51A*, *cyp51B* or *cyp51C*. This observation strongly suggests the involvement of alternative resistance mechanisms, such as efflux pump overexpression (e.g., ABC transporters), regulatory mutations, or post-transcriptional adaptations, which warrant further investigation.

Several limitations should nevertheless be acknowledged, including the relatively limited number of resistant isolates analyzed and the absence of functional validation experiments such as targeted mutagenesis or gene replacement assays. Therefore, the proposed contribution of the I285V substitution and *cyp51C* overexpression to azole resistance should be interpreted as associative rather than definitive.

Taken together, our findings support the hypothesis that *cyp51C* alterations may contribute to reduced azole susceptibility in *A. flavus* through a complex interplay between transcriptional regulation, structural variation, and potentially additional compensatory mechanisms. The absence of uniform *cyp51* overexpression among resistant isolates, particularly in strain TN-7, further emphasizes the multifactorial nature of azole resistance in this species. Although functional validation experiments were not performed, the convergence of transcriptional, mutational, phenotypic, and structural modeling data supports a potential contribution of *cyp51C* alterations to reduced azole susceptibility in *A. flavus*. These findings may contribute to improving molecular surveillance strategies for azole-resistant *A. flavus* isolates and support the future development of molecular markers for early detection of emerging resistance.

In conclusion, our findings suggest that azole resistance in the *A. flavus* isolates investigated in this study appears to be associated with multiple molecular mechanisms, including indirectly acting point mutations and the overexpression of *cyp51A*, *cyp51B* and *cyp51C* genes. This observation is consistent with recent studies reporting that resistance in non-*fumigatus Aspergillus* species cannot be attributed to a single dominant mechanism but rather reflects the interplay of multiple genetic and regulatory factors [27,30].

Furthermore, the overexpression of *cyp51C* independently of *cyp51A* and *cyp51B* appears to play a contributory, and potentially modulatory, role in the development of the azole-resistant phenotype in *A. flavus*. This finding supports emerging evidence from recent studies highlighting the species-specific involvement of *cyp51C* in azole susceptibility and reinforces the importance of considering cyp51C as a relevant molecular marker when investigating resistance mechanisms in *A. flavus*.

Overall, these results emphasize the need for integrated molecular approaches combining gene expression profiling, sequence analysis, and functional validation to accurately characterize azole resistance in *A. flavus*, particularly in light of the increasing clinical incidence of azole-resistant isolates. In addition, improved understanding of resistance-associated molecular mechanisms may contribute to the development of molecular surveillance strategies, facilitate the early detection of emerging resistant strains, and ultimately support more effective antifungal management of aspergillosis.

Therefore, the effective detection and management of *A. flavus* infections should account for this combinatorial model, integrating gene expression profiling alongside mutation analysis to provide a more accurate prediction of treatment outcomes and to mitigate the risk of therapeutic failure.

## Figures and Tables

**Table 1:** The sequences of primers and probes used in RT-qPCR.

Gene	Primers and Probes	
*Cyp51C*	F	5′-TCCCACCACAGTGTACCTG-3′
	R	5′-TAGTGATCTCCGCCATAGCC-3′
	Probe	FAM-ATGATTGCCCCAATTCCAAG-MGB
*ACT1*	F	5′-AGTCACACACGTGGTTCCAA-3′
	R	5′-TTGATGTCGCGCACTATCTC-3′
	Probe	TET-GGCCATAGCTTCACCACATC-MGB

**Table 2:** The sequences of primers used in PCR sequencing.

Gene	Primers	
*Cyp51C*	AP1F	5′-GCCCTGAATGTCACCTATCA3′
	AP1R	5′-GCCCAGGGTAGCATGAAGTT-3′
	AP2F	5′-GCAGTGCACCTACAAGAACG-3′
	AP2R	5′-AGGTGACGCACCATAGTGG-3′
	AP3F	5′-GGCTATGGCGGAGATCACTA-3′
	AP3R	5′-TGTATTGTAGCGGAGCCAGA-3′

**Table 3:** Antifungal Susceptibility, Relative Gene Expression, Gene Copy Number, and Point Mutations of the *cyp51C* Gene in *A. flavus*.

		Isolates	IT		POS		RNA Relative Quantification	DNA Relative Quantification	*cyp51C*										Cyp51C Mutated Amino Acid			
			MIC	R/S	MIC	R/S	*cyp51C*	*cyp51C*	Mutation Ponctuelle										*Cyp51C*			
Patient 1	AI	TN-1	0.125	S	0.125	S	2.9	1.712	T-C*(161)	C-G*(174)	No mutation	No mutation	T-G*(788)	No mutation	No mutation	No mutation	No mutation	No mutation	M-T*(54)	S-A*(240)	No mutation	No mutation
		TN-2	0.125	S	0.125	S	0.605	0.848	T-C*(161)	C-G*(174)	No mutation	No mutation	T-G*(788)	No mutation	No mutation	No mutation	No mutation	No mutation	M-T*(54)	S-A*(240)	No mutation	No mutation
		TN-3	0.125	S	0.125	S	1.153	0.145	T-C*(161)	C-G*(174)	No mutation	No mutation	T-G*(788)	No mutation	No mutation	No mutation	No mutation	No mutation	M-T*(54)	S-A*(240)	No mutation	No mutation
Patient 2	AI	TN-4	0.125	S	0.19	S	4.242	0.227	T-C*(161)	C-G*(174)	No mutation	No mutation	T-G*(788)	No mutation	No mutation	No mutation	No mutation	No mutation	M-T*(54)	S-A*(240)	No mutation	No mutation
		TN-5	0.125	S	0.064	S	0.615	0.648	T-C*(161)	C-G*(174)	No mutation	No mutation	T-G*(788)	No mutation	No mutation	No mutation	No mutation	No mutation	M-T*(54)	S-A*(240)	No mutation	No mutation
		TN-6	0.032	S	0.094	S	0,173	0.002	T-C*(161)	C-G*(174)	No mutation	No mutation	T-G*(788)	No mutation	No mutation	No mutation	No mutation	No mutation	M-T*(54)	S-A*(240)	No mutation	No mutation
Patient 3	AI	TN-7	1.5	R	0.125	S	1.811	0.21	T-C*(161)	C-G*(174)	G-A*(757)	G-A*(781)	T-G*(788)	G-A*(830)	A-G*(923)	T-C*(946)	T-A*(964)	C-T*(1228)	M-T*(54)	S-A*(240)	D-N *(254)	I-V*(285)
		TN-8	0.5	S	0.094	S	1.399	0.851	T-C*(161)	C-G*(174)	No mutation	No mutation	T-G*(788)	No mutation	No mutation	No mutation	No mutation	No mutation	M-T*(54)	S-A*(240)	No mutation	No mutation
Patient 4	AI	TN-9	0.75	S	0.125	S	2.411	5.398	T-C*(161)	C-G*(174)	G-A*(757)	G-A*(781)	T-G*(788)	G-A*(830)	A-G*(923)	T-C*(946)	T-A*(964)	C-T*(1228)	M-T*(54)	S-A*(240)	D-N*(254)	I-V*(285)
		TN-10	0.38	S	0.064	S	4.703	7.281	T-C*(161)	C-G*(174)	No mutation	No mutation	T-G*(788)	No mutation	No mutation	No mutation	No mutation	No mutation	M-T*(54)	S-A*(240)	No mutation	No mutation
Patient 5	AI	TN-11	0.5	S	0.125	S	2.383	0.635	T-C*(161)	C-G*(174)	No mutation	No mutation	T-G*(788)	No mutation	No mutation	No mutation	No mutation	No mutation	M-T*(54)	S-A*(240)	No mutation	No mutation
		TN-12	0.125	S	0.125	S	4.377	1.51	T-C*(161)	C-G*(174)	No mutation	No mutation	T-G*(788)	No mutation	No mutation	No mutation	No mutation	No mutation	M-T*(54)	S-A*(240)	No mutation	No mutation
Patient 6	AI	TN-13	0.75	S	0.19	S	0.3	0.27	T-C*(161)	C-G*(174)	G-A*(757)	G-A*(781)	T-G*(788)	G-A*(830)	A-G*(923)	T-C*(946)	T-A*(964)	C-T*(1228)	M-T*(54)	S-A*(240)	D-N*(254)	I-V*(285)
		TN-14	0.5	S	0.125	S	1.065	0.056	T-C*(161)	C-G*(174)	No mutation	No mutation	T-G*(788)	No mutation	No mutation	No mutation	No mutation	No mutation	M-T*(54)	S-A*(240)	No mutation	No mutation
		TN-15	1	R	0.19	S	5.354	1.053	T-C*(161)	C-G*(174)	G-A*(757)	G-A*(781)	T-G*(788)	G-A*(830)	A-G*(923)	T-C*(946)	T-A*(964)	C-T*(1228)	M-T*(54)	S-A*(240)	D-N*(254)	I-V*(285)
		TN-16	1	R	0.19	S	4.947	3.952	T-C*(161)	C-G*(174)	G-A*(757)	G-A*(781)	T-G*(788)	G-A*(830)	A-G*(923)	T-C*(946)	T-A*(964)	C-T*(1228)	M-T*(54)	S-A*(240)	D-N*(254)	I-V*(285)
Patient 7	AI	TN-17	0.75	S	0.125	S	2.021	1.781	T-C*(161)	C-G*(174)	G-A*(757)	G-A*(781)	T-G*(788)	G-A*(830)	A-G*(923)	T-C*(946)	T-A*(964)	C-T*(1228)	M-T*(54)	S-A*(240)	D-N*(254)	I-V*(285)
		TN-18	0.5	S	0.125	S	0.421	0.783	T-C*(161)	C-G*(174)	No mutation	No mutation	T-G*(788)	No mutation	No mutation	No mutation	No mutation	No mutation	M-T*(54)	S-A*(240)	No mutation	No mutation
		TN-19	0.75	S	0.125	S	1.054	0.07	T-C*(161)	C-G*(174)	No mutation	No mutation	T-G*(788)	No mutation	No mutation	No mutation	No mutation	No mutation	M-T*(54)	S-A*(240)	No mutation	No mutation
		TN-20	0.38	S	0.064	S	6.352	0.074	T-C*(161)	C-G*(174)	No mutation	No mutation	T-G*(788)	No mutation	No mutation	No mutation	No mutation	No mutation	M-T*(54)	S-A*(240)	No mutation	No mutation
Patient 8	AI	TN-21	0.38	S	0.125	S	0.385	0.298	T-C*(161)	C-G*(174)	No mutation	No mutation	T-G*(788)	No mutation	No mutation	No mutation	No mutation	No mutation	M-T*(54)	S-A*(240)	No mutation	No mutation
Patient 9	AI	TN-22	0.25	S	0.125	S	0.369	0.786	T-C*(161)	C-G*(174)	No mutation	No mutation	T-G*(788)	No mutation	No mutation	No mutation	No mutation	No mutation	M-T*(54)	S-A*(240)	No mutation	No mutation
Patient 10	AI	TN-23	0.38	S	0.125	S	0.122	0.916	T-C*(161)	C-G*(174)	No mutation	No mutation	T-G*(788)	No mutation	No mutation	No mutation	No mutation	No mutation	M-T*(54)	S-A*(240)	No mutation	No mutation
Patient 11	AI	TN-24	0.38	S	0.125	S	0.3	0.04	T-C*(161)	C-G*(174)	No mutation	No mutation	T-G*(788)	No mutation	No mutation	No mutation	No mutation	No mutation	M-T*(54)	S-A*(240)	No mutation	No mutation
		TN-25	0.75	S	0.125	S	0.668	0.829	T-C*(161)	C-G*(174)	No mutation	No mutation	T-G*(788)	No mutation	No mutation	No mutation	No mutation	No mutation	M-T*(54)	S-A*(240)	No mutation	No mutation
		TN-26	0.75	S	0.125	S	5.215	3.561	T-C*(161)	C-G*(174)	G-A*(757)	G-A*(781)	T-G*(788)	G-A*(830)	A-G*(923)	T-C*(946)	T-A*(964)	C-T*(1228)	M-T*(54)	S-A*(240)	D-N*(254)	I-V*(285)
Patient 12	AI	TN-27	0.25	S	0.125	S	1.99	0.735	T-C*(161)	C-G*(174)	G-A*(757)	G-A*(781)	T-G*(788)	No mutation	No mutation	No mutation	No mutation	No mutation	M-T*(54)	S-A*(240)	No mutation	No mutation
		TN-28	0.25	S	0.125	S	2.89	0.632	T-C*(161)	C-G*(174)	No mutation	No mutation	T-G*(788)	No mutation	No mutation	No mutation	No mutation	No mutation	M-T*(54)	S-A*(240)	No mutation	No mutation
		TN-29	0.5	S	0.094	S	0.068	1.238	T-C*(161)	C-G*(174)	No mutation	No mutation	T-G*(788)	No mutation	No mutation	No mutation	No mutation	No mutation	M-T*(54)	S-A*(240)	No mutation	No mutation
Patient 13	AI	TN-30	0.5	S	0.094	S	2.022	6.538	T-C*(161)	C-G*(174)	No mutation	No mutation	T-G*(788)	No mutation	No mutation	No mutation	No mutation	No mutation	M-T*(54)	S-A*(240)	No mutation	No mutation
		TN-31	1.5	R	0.75	R	2.56	1.168	T-C*(161)	C-G*(174)	No mutation	No mutation	T-G*(788)	No mutation	No mutation	No mutation	No mutation	No mutation	M-T*(54)	S-A*(240)	No mutation	No mutation
		TN-32	0.5	S	1	R	3.649	1.724	T-C*(161)	C-G*(174)	G-A*(757)	G-A*(781)	T-G*(788)	G-A*(830)	A-G*(923)	T-C*(946)	T-A*(964)	C-T*(1228)	M-T*(54)	S-A*(240)	D-N *(254)	I-V*(285)
Patient 14	AI	TN-33	1	R	0.75	R	5.474	0.866	T-C*(161)	C-G*(174)	G-A*(757)	G-A*(781)	T-G*(788)	G-A*(830)	A-G*(923)	T-C*(946)	T-A*(964)	C-T*(1228)	M-T*(54)	S-A*(240)	D-N *(254)	I-V*(285)
		TN-34	0.38	S	0.064	S	0.885	0.052	T-C*(161)	C-G*(174)	No mutation	No mutation	T-G*(788)	No mutation	No mutation	No mutation	No mutation	No mutation	M-T*(54)	S-A*(240)	No mutation	No mutation

* Punctual mutation; S—susceptible; R—resistant; MIC in μg/mL.

**Table 4:** The amino acids of the ligand binding site.

Protein	Posaconazole	Itraconazole
Cyp51C	**Y106**, **L109**, **T110**, **F114**, **Y120**, **F213**, **P215**, **T288**, **M291**, **A292**, **S296**, **I363**, **L367**, **L493**	**T49**, **Y52**, **G53**, **Y106**, **F114**, **V119**, **Y120**, **F213**, **P215**, **T288**, **L289** **A292**, **S296**, **I363**, **H364**, **S365**, **L367**, **S491**, **A492**, **L493**

Residues highlighted in red indicate amino acids directly interacting with azole compounds in the predicted binding site, while residues in black correspond to surrounding residues within the binding pocket.

## Data Availability

The original contributions presented in this study are included in the article. Further inquiries can be directed to the corresponding author.
